# A Review of the Epigenetic Clock: Emerging Biomarkers for Asthma and Allergic Disease

**DOI:** 10.3390/genes14091724

**Published:** 2023-08-29

**Authors:** Denitsa Vasileva, Celia M. T. Greenwood, Denise Daley

**Affiliations:** 1 Centre for Heart Lung Innovation, University of British Columbia and Saint Paul’s Hospital, Vancouver, BC V6Z 1Y6, Canada; denitsa.vasileva@hli.ubc.ca; 2 Lady Davis Institute for Medical Research, Montreal, QC H3T 1E2, Canada; celia.greenwood@mcgill.ca; 3 Department of Epidemiology, Biostatistics and Occupational Health, McGill University, Montreal, QC H3A 0G4, Canada; 4 Gerald Bronfman Department of Oncology, McGill University, Montreal, QC H3A 0G4, Canada; 5 Department of Human Genetics, McGill University, Montreal, QC H3A 0G4, Canada; 6 Department of Medicine, Respiratory Division, University of British Columbia, Vancouver, BC V6T 1Z3, Canada

**Keywords:** allergy, asthma, biomarker, epigenetic clock

## Abstract

DNA methylation (DNAm) is a dynamic, age-dependent epigenetic modification that can be used to study interactions between genetic and environmental factors. Environmental exposures during critical periods of growth and development may alter DNAm patterns, leading to increased susceptibility to diseases such as asthma and allergies. One method to study the role of DNAm is the epigenetic clock—an algorithm that uses DNAm levels at select age-informative Cytosine-phosphate-Guanine (CpG) dinucleotides to predict epigenetic age (EA). The difference between EA and calendar age (CA) is termed epigenetic age acceleration (EAA) and reveals information about the biological capacity of an individual. Associations between EAA and disease susceptibility have been demonstrated for a variety of age-related conditions and, more recently, phenotypes such as asthma and allergic diseases, which often begin in childhood and progress throughout the lifespan. In this review, we explore different epigenetic clocks and how they have been applied, particularly as related to childhood asthma. We delve into how in utero and early life exposures (e.g., smoking, air pollution, maternal BMI) result in methylation changes. Furthermore, we explore the potential for EAA to be used as a biomarker for asthma and allergic diseases and identify areas for further study.

## 1. Introduction

**Figure 1:**
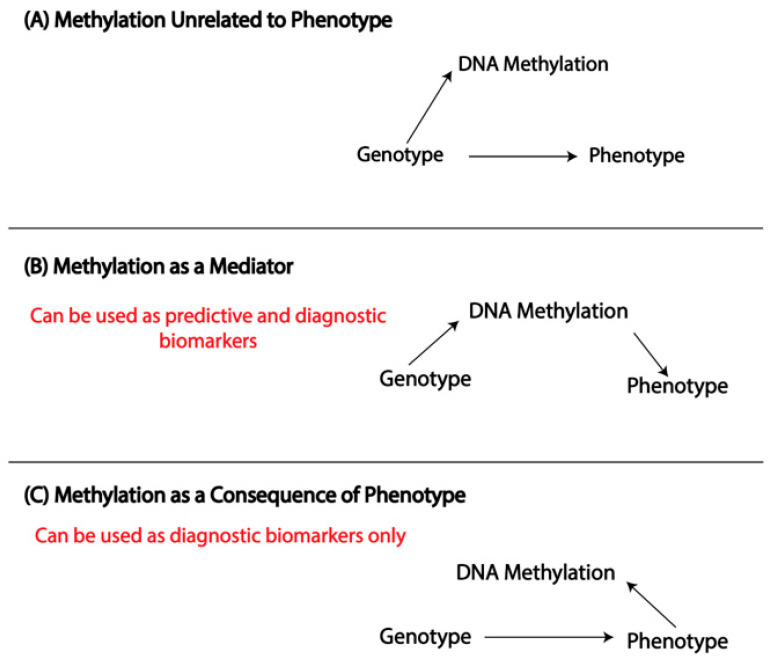
Causality and biomarkers. Epigenetic changes such as DNA methylation (DNAm) can be (**A**) unrelated to a phenotype, (**B**) a cause, or (**C**) a consequence. (**A**) DNA methylation is affected by genotype, which also independently causes a phenotype. (**B**) Genotype causes phenotype, mediated by DNA methylation (a mediator). (**C**) A genotype causes a phenotype, which, in turn, affects DNA methylation (reverse causality). In both (**B**,**C**), DNA methylation can be used as a diagnostic biomarker, but it can only be used as a predictive biomarker in (**B**).

DNA methylation (DNAm) is a dynamic epigenetic modification that refers to the bonding of a methyl (CH3) group to the fifth carbon of a Cytosine base to form 5-methyl-Cytosine [1]. This process primarily occurs at Cytosine-phosphate-Guanine (CpG) dinucleotides [2]. Methylation levels can be altered by environmental factors (e.g., smoking [3], pesticide exposures [4]), disease (including asthma [5]), cell type [6], sex [7], and age [2]) and may play a causal or intermediary role in the development of disease (Figure 1). DNAm can be used to characterize the relationship between gene-environment interactions and disease.

Many complex phenotypes—including asthma and allergic disease—have an age-dependent presentation. The role of epigenetics in these conditions can be studied by exploiting the relationship between DNA methylation and aging. Consistent universal patterns of methylation change due to age have been identified [2,8,9,10], resulting in the development of epigenetic clocks [11]. These are mathematical algorithms that use methylation levels at select CpG sites to calculate epigenetic age [12] as a measure of biological aging. The epigenetic clock provides a concise summary of DNAm at CpG sites across the genome.

Differences between an individual’s epigenetic and chronological ages may point to deviation, either due to disease or exposure, from the expected patterns of age-related methylation. Epigenetic age acceleration (EAA, epigenetic age > chronological age) has shown utility as a biomarker in age-related conditions [13,14,15] and has been linked to pediatric asthma and allergy [16,17]. Due to the labile nature of the modification, it is difficult to establish whether changes in methylation are a cause or a result of disease (Figure 1). A predictive biomarker requires a causal relationship (Figure 1B). However, a diagnostic biomarker for complex diseases such as asthma necessitates only a change in methylation that is specifically associated with the phenotype (Figure 1A).

**Figure 2:**
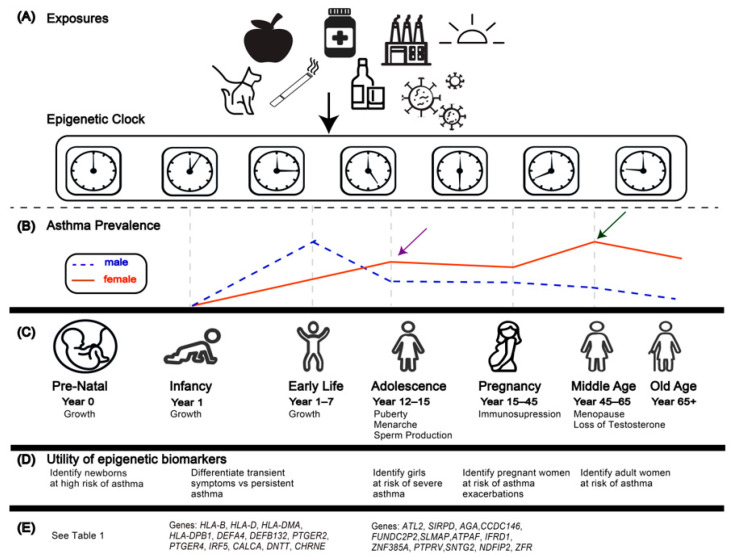
The epigenetic clock and asthma across the life course. (**A**) illustrates different environmental exposures that may alter epigenetic aging. (**B**) is an illustrative representation of the proportion of male vs. female asthmatics over the life stages. Arrows indicate key changes in sex-specific patterns of prevalence as extrapolated from [30,31,32] and are for illustrative purposes only. (**C**) shows the different life stages. During periods of significant hormone changes (e.g., puberty, pregnancy, and menopause), DNA is particularly vulnerable to alteration by environmental exposures. (**D**) demonstrates the utility of epigenetic asthma biomarkers at different time points. (**E**) shows the key genes undergoing changes in methylation during development [33,34,35].

A key question is whether epigenetic age acceleration is set prenatally and impacts disease risk in later life. This aligns with the Developmental Origins of Health and Disease (DOHaD) [18] hypothesis that exposures during the first 1000 days of life lead to changes in methylation that contribute to disease in adulthood. This premise has led to studies of the impact of gestational and early life exposures on epigenetic aging and on the development of asthma and allergic disease (Figure 2A). Differential methylation has been observed in cord blood due to in utero exposures such as elevated maternal BMI [19], air pollution [20], and the repeatedly replicated effects of maternal smoking [21,22,23,24] (Table 1).

Asthma is a complex, heterogeneous phenotype with age- and sex-specific prevalence patterns [31]. Childhood asthmatics are predominately male (65%), while 65% of adult asthmatics are female (Figure 2B) [31]. There are many phenotypes and endotypes of asthma. Allergic asthma (i.e., asthma present in conjunction with an IgE-mediated allergic disease) accounts for ~90% of childhood cases and ~50% of adult cases [36]. Asthma is often thought to be driven by in-utero and early-life influences. Exposure to pollutants such as bisphenols (BPs) during pregnancy has been associated with asthma in adolescent girls [37], while maternal stress [38] and prenatal smoking [39] have been linked to childhood asthma. Epigenetic modifications have been extensively associated with asthma and allergic diseases. These associations have been previously reviewed [40,41] and are not the focus of this paper. It is important to consider that associations between exposures, epigenetic modifications, and disease phenotypes do not equate with demonstrating causality or pathogenesis, as these epigenetic modifications may be secondary to the development of the phenotype (Figure 1). We emphasize the potential of the epigenetic clock as a cumulative indicator of both DNAm across the genome and epigenetic modifications due to exposure or disease, independent of disease etiology, as a diagnostic biomarker in asthma. Currently, asthma diagnosis is based largely on symptom presentation, which may be unreliable, and a diagnostic molecular biomarker would be of clinical utility. The epigenetic clock can be used to identify early-life exposures that affect methylation [42], paving the way for epigenetic diagnostic biomarkers for asthma.

## 2. Purpose

Provide an overview of current epigenetic clocks, explore their utility in early childhood, and highlight their applications as potential biomarkers for asthma and allergic disease.

## 3. Overview of DNA Methylation

About 70% of CpG sites in the human genome are methylated [43]. CpGs are concentrated in CpG islands (CGIs)—regions > 200 base pairs—where C-G dinucleotides make up more than 50% of the sequence [1,44]. Islands house the promoters of ~70% of human genes [44,45]. Methylation in these areas is linked to repressed gene expression, but the relationship is mediated by CpG density [46].

Different technologies have been developed for assessing DNA methylation, but arrays and sequencing protocols form the basis of the literature. Three arrays have been predominantly used in human studies: the legacy Illumina Human Methylation 27 Bead Chip (Illumina, Inc., San Diego, CA, USA) [47], the Illumina Human Methylation 450 Bead Chip (Illumina, Inc., San Diego, CA, USA) [48], and the Illumina Methylation EPIC Bead Chip array (Illumina, Inc., San Diego, CA, USA) [49]. Each features progressive expansion of coverage and increased representation of different genomic regions, with the EPIC assaying ~30× more sites than the 27 K, especially outside of islands [49].

Common changes in DNA methylation over the lifespan across individuals have been identified by multiple studies [50,51,52]. Cord blood generally displays low levels of methylation across the genome [53,54] followed by a rapid increase in early life [34,54], and a gradual loss in later years [2]. CpG sites linked to embryonic developmental genes gain methylation in childhood, while regions related to immune processes lose methylation [33]. For example, genes located in Major Histocompatibility Complex (MHC) classes I and II [33,55]—in particular *HLA-B*, *HLA-C*, *HLA-DMA*, and *HLA-DPB1*—become demethylated with age. MHC I and II play a crucial role in the immune response and have been implicated in asthma and allergic disease (Figure 2E) [56,57].

There are two key components to the immune system: innate and adaptive immunity. MHC complexes are part of the adaptive immune system and are involved in recognizing and destroying pathogens [58]. Innate immunity is present in the fetus and at birth but is subdued to tolerate the stress of fetal development [59]. Adaptive immunity develops throughout the lifespan, with T cells playing a key role. Helper T cells are heavily involved in asthma and allergic diseases. They can be further differentiated into Th1 or Th2 cells. Th2 cells stimulate the production of antibodies and have been linked to an increased immunoglobulin E (IgE) response in atopy [60] and asthma [61]. Fetal and neonatal T cells differ significantly from adult cells. Environmental exposures may activate fetal/neonatal T cells, resulting in a Th2 immune response [59] (Box 1). The role of these cells in asthma severity may be sex-specific, as Th2 cell abundance has been shown to be correlated to asthma symptom severity in adult women but not men [62]. In addition, Zhang et al. found that changes in methylation within Th2 pathway genes between the ages of 10 and 18 increase the risk of asthma development in girls [63].

DNA methylation fluctuates during childhood at a rate three to four times greater than in adulthood [64]. These early changes might follow a logarithmic, rather than linear, pattern with age [64,65,66]. The accumulation of changes with time leads to larger inter-individual variability in methylation with age, a process known as epigenetic drift [67,68]. It has been postulated that aging may be a process of ‘memorizing’ environmental exposures [69].

Box 1 **Molecular mechanisms of sex hormones, DNAm, and asthma.**

The molecular mechanism behind the sex-specificity of asthma has not been fully elucidated. Adult women with severe asthma have higher levels of inflammation (e.g., circulating Th2 cells) compared to males [62]. Previous animal work has shown that the sex hormone estrogen increases inflammation in female mice, while androgens [70] (e.g., testosterone) decrease it in male mice. In humans, estrogen is linked to increased differentiation of Th2 cells by influencing the expression of *CRTH2* [62] (a receptor on Th2 cells). DNA methylation may also play a role in this mechanism. Exposure to environmental estrogens leads to a decrease in methylation at H3K27me3 (histone 3) in T cells [71]. Moreover, in mouse models, these exposures can lead to a loss of methylation in helper T cells and a subsequent increase in Th1 and Th2 cells, persisting across generations [72].

## 4. Epigenetic Clocks

Epigenetic clocks exploit the reproducible relationship between methylation at specific CpGs and age to calculate epigenetic age. CpG sites are usually selected using penalized linear regression methods (e.g., elastic net regression) [12,73]. Discrepancies between epigenetic and chronological age can highlight changes in cell or tissue function [12]. Epigenetic age acceleration has been associated with exposures such as tobacco [74] and implicated in disease [13,15] and time to mortality [14,75]. There is some evidence that maternal exposures (e.g., smoking) are associated with acceleration in the offspring [42]. Meanwhile, epigenetic age deceleration (EAD, epigenetic age < chronological age) has been connected to exercise [76]. The utility of epigenetic clocks as biomarkers for complex diseases is of biological significance and is the focus of this review.

Epigenetic clocks have mostly been studied in association with adult diseases. To be useful in pediatric conditions, they need to accurately model the dynamic nature of age-related DNA methylation during early life. Furthermore, current linear methods do not account for any non-additive interactions between CpG sites. This limitation can be addressed by using non-linear methods to build epigenetic clocks.

## 5. Epigenetic Clock Training Metrics

There are two types of epigenetic clocks: first- and second-generation clocks. First-generation clocks use raw or log-transformed chronological age [65] as the dependent variable, whereas a composite measure of aging is the dependent variable in second-generation clocks. This measure includes biological aging proxies, such as heart function markers, and chronological age [77].

## 6. First-Generation Epigenetic Clocks

First-generation clocks have been used in the bulk of epigenetic clock research. Despite being trained solely on chronological age, the epigenetic age acceleration calculated by these clocks has been implicated in the incidence of disease [78]. First-generation clocks can be further classified into single- and multi-tissue clocks. In this review, we discuss the clocks that have broad utility (e.g., the Hannum clock [79], the Horvath pan-tissue [65] and Skin and Blood clocks [80], and the Pediatric-Buccal-Epigenetic (PedBE) clock [81]. The features of other clocks tailored to narrower use cases are summarized in Table 2. The accuracy of first-generation epigenetic clocks is assessed in relation to chronological age, usually using Absolute Error (AE = |epigenetic-chronological age|) or Pearson’s correlation coefficient (*r*). Chronological and epigenetic age are correlated, but the deviation between the two has been shown to be informative of ‘biological capacity’ (e.g., physical fragility, disease susceptibility) in adults [82].

**Figure 3:**
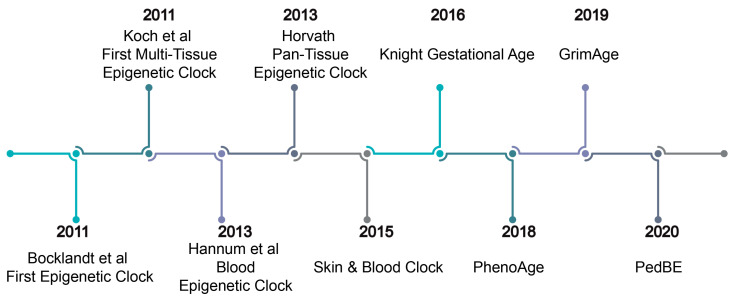
A timeline of key epigenetic clocks. A timeline of the development of key epigenetic clocks presented in this review [65,77,79,80,81,83,86,87,90].

The first clock was developed in 2011 by Bocklandt et al. [83] using saliva samples (Figure 3). Due to the tissue specificity of methylation, it was not generalized to other tissue types (Table 2) [83]. Shortly thereafter, the Hannum [79] clock—a blood epigenetic clock—was published. Using the 450 K array (Illumina Inc., San Diego, CA, USA) data from 656 samples (482 in the training set and 174 in the testing set) of whole blood (age range: 19–101 years), this clock was developed in stages [79]. First, ~70,000 age associated autosomal CpG sites were identified. Furthermore, elastic net regression with bootstrapping was performed to build a clock of 71 CpGs [79] (Table 2). The Hannum clock demonstrated low accuracy in pediatric samples [89], likely due to only using adult samples in model development [89].

## 7. First-Generation Multi-Tissue Epigenetic Clocks

Teschendorff et al. (2010) [91] described a set of 69 CpGs with age-associated increases in methylation in both blood and epithelial tissue, showing a multi-tissue signature of aging. Koch et al. used four different tissue types (Table 2 and Figure 3) to develop the first multi-tissue clock [86]. Two multi-tissue clocks have been developed by Horvath et al.: the pan-tissue [65] and Skin and Blood clocks [80] (Figure 3).

The pan-tissue Horvath clock [65] forms the backbone of epigenetic aging studies. It was developed using 8000 samples (from 51 healthy tissues) of Illumina 27 K and 450 K data, divided into training and validation cohorts. Elastic net regression with 10-fold cross validation was performed on the methylation values of 21,369 CpG sites with a log-transformed version of chronological age as the dependent variable. This regression yielded a clock of 353 CpG sites with a median absolute error between predicted and reported ages of 3.6 years in the validation cohort [65]. The pan-tissue Horvath clock has shown a high correlation with chronological age, even when applied to data from the Illumina 850 K EPIC (Illumina Inc., San Diego, CA, USA) array (missing 19/353 sites) [92]; in addition, it demonstrates robustness to changes in cell type composition [89]. However, this clock has several drawbacks. For example, repeated underestimation of epigenetic age in older individuals and variable accuracy in children with cell type composition [89] have both been observed [93]. Moreover, this clock was mostly developed using adult samples and may not contain the CpG sites associated with early developmental processes, thus limiting its utility as a biomarker in pediatric conditions.

The Horvath Skin and Blood clock aimed to improve the accuracy of the pan-tissue clock in fibroblasts [80]. It consists of 391 CpG sites and was built using the same methodology as the pan-tissue clock but with Illumina 450 K or 850 K array (Illumina Inc., San Diego, CA, USA) data from buccal cells, fibroblasts, keratinocytes, endothelial cells, blood, and saliva [80]. This clock is more accurate than the Horvath pan-tissue clock and Hannum clock in blood samples (median AE = 2.5 vs. 3.7 and 5.1 years) [80].

## 8. Pediatric Epigenetic Clock

As the field of epigenetic aging has broadened to study the effects of childhood exposures, pediatric epigenetic clocks have been developed. The most prominent childhood clock is the PedBE [81] clock—a 94-CpG buccal epithelial cell clock developed using exclusively pediatric samples (age range: 0.17–19.47 years). Elastic net regression was performed on Illumina 850 K array data from 1032 children to identify clock CpGs and their weights. PedBE’s performance was then evaluated in an independent set of 689 buccal samples (age range: 0.01–19.96 years) [81], where it had a median absolute error of 0.35 years, demonstrating greater accuracy for that age group compared to the pan-tissue Horvath clock [81]. However, when applied to an independent set of blood samples *(n* = 134), the PedBE clock had a higher median absolute error than the Horvath pan-tissue clock (3.26 vs. 0.57 years) [81]. This performance discrepancy (blood vs. buccal samples) was expected as methylation patterns are cell and tissue type specific.

## 9. Gestational Age Clocks

The cell type specificity of methylation has complicated the use of the epigenetic clock to study prenatal environmental exposures [87], as the cellular composition of cord blood is distinct from that of venous blood. Hence, clocks developed using venous blood may be unsuitable for epigenetic gestational age predictions. The Horvath pan-tissue clock incorporated cord blood samples in its training set but set their age at “0” [65]. This may lead to lower accuracy in neonatal blood samples as it does not account for gestational age. To address this, gestational epigenetic clocks have been developed [26,87,88], as summarized in Table 2 and Figure 3. Our understanding of the relationship between maternal exposures and methylation has expanded rapidly. However, there is inconsistency between some studies using gestational age (estimated through either the last menstrual period or ultrasound methods) [21] and others using epigenetic gestational age. Epigenetic gestational age acceleration can provide insight into the role of methylation in traits in infancy.

## 10. Second-Generation Epigenetic Clocks

First-generation epigenetic clocks are useful in the study of phenotypes and epigenetic aging, but because they were trained exclusively on chronological age [11], they may not select the most health informing CpG sites. Second-generation epigenetic clocks are developed using variables indicative of health status (e.g., five plasma proteins and smoking status) in addition to chronological age. They aim to improve the performance of the first-generation clocks in predicting disease development and mortality. PhenoAge [77] and GrimAge [90] (Table 3) are second-generation clocks and assess time-to-death more accurately than first-generation clocks. However, the clinical markers used to generate a composite “biological age” value make these clocks difficult to implement, as detailed health data may not be available. In addition, the markers used (e.g., albumin and creatinine) may be relevant to aging in older adults but may not be informative in pediatric samples; thus, child-specific second-generation clocks may be needed.

## 11. Metrics of Epigenetic Age Acceleration

Epigenetic age acceleration underlines the potential use of epigenetic clocks as biomarkers. The most frequently used approaches to assess epigenetic age acceleration are: (1) Intrinsic Epigenetic Age Acceleration (IEAA) and (2) Extrinsic Epigenetic Age Acceleration (EEAA), as they both consider the cell-specificity of DNA methylation patterns (Box 1). IEAA calculates accelerated aging independent of age-related changes in blood cell type composition. EEAA includes both methylation changes due to age and those due to age-related changes in cell type composition [94]. Other methods for assessing age acceleration are the difference between epigenetic and chronological age and the residual of the regression of epigenetic on chronological age (AgeAccel) (Box 2).

Box 2 **The epigenetic clock and epigenetic age acceleration.**

Epigenetic age acceleration characterizes the relationship between the calculated epigenetic age and the reported chronological age. It can be assessed using the following methods:

1. Intrinsic Epigenetic Age Acceleration (IEAA)—epigenetic age acceleration independent of cell type composition. This captures the “intrinsic” process of aging and should be universal, regardless of cell and tissue type. This metric is calculated by extracting the residuals of the linear regression:
  Horvath Epigenetic Age ~ Chronological age+Naive CD8 cells+Exhausted CD8 cells+plasmablasts+Natural killer cells+monocytes+granulocytes

Usage: IEAA is not highly correlated with external factors [95] and should be used when interested in changes in pure cellular aging.

2. Extrinsic Epigenetic Age Acceleration (EEAA)—a measure of age acceleration including both intrinsic age-related processes and changes in cell type, calculated in two steps:
  - Enhanced Hannum epigenetic age is the weighted average of epigenetic age predicted by the Hannum clock and a combination of cell types. The weights are determined using a correlation between cell type and chronological age.
  - The second step is a regression of enhanced Hannum age on chronological age
    Enhanced Hannum Epigenetic Age ~ Chronological Age.

Usage: EEAA captures both changes in the epigenetic clock and in cell type [95] (immune system aging) and is highly correlated with external factors. EEAA is based on the Hannum clock, which is not accurate for children.

3. Age Acceleration (AgeAccel) [96]—a measure obtained by extracting the residuals of the linear regression of epigenetics on chronological age without accounting for cell type: *Epigenetic* *Age* ~ *Chronological* *Age*.

Usage: This method is the most frequently used but does not account for the age-associated changes in cell type proportion that affect methylation.

## 12. Applications of the First-Generation Epigenetic Clocks to Asthma and Allergic Disease

Asthma presents a test case for the utility of the epigenetic clock in studying prenatal and childhood traits and exposures over the lifespan. This condition often starts in the early years. Diagnosis, especially of pediatric asthma, is performed by exclusion, relying on a diverse clinical presentation.

There is a well-established age-related pattern to asthma development. Childhood asthma is part of the atopic march, which has an age-specific course [97]—beginning in infancy with eczema/atopic dermatitis, progressing to infant food allergies, then asthma and allergic rhinitis. In adolescence, there is an unexplained switch in the sex-specific prevalence of asthma [31] (Figure 2C and Box 2). Adult asthmatics, predominantly female, are prone to severe asthma, particularly after menopause [31] (Box 2). Diagnosis by presentation alone cannot distinguish the different asthma phenotypes and endotypes or differentiate between adult, pediatric, and severe vs. non-severe asthma.

A similar lack of a diagnostic test is seen in allergic diseases. Skin prick tests (SPT) are frequently used, but positive results are not always indicative of an allergic reaction. The gold standard test for food allergies—the oral food challenge—carries risk and is only performed in specialized settings [98].

Genetic information has been used to assess asthma risk. Genetic variants account for ~61–75% of susceptibility to asthma [99,100]. Genome-wide association studies have demonstrated associations between genes in the Human Leukocyte Antigens (HLA) region and asthma and allergic disease [101,102]. The remaining ~25–40% of the risk may be due to environmental factors, with effects dependent on age and sex. Differential methylation at CpG sites has been reported in both child and adult asthmatics [103,104].

The inclusion of epigenetic information can explain heterogeneity within the asthma phenotype and is necessary for the development of a diagnostic biomarker.

For example, respiratory syncytial virus (RSV) infection in infancy is associated with a higher risk of asthma [73]. Viral infection can have either a punitive or protective effect, depending on age and viral subtype [105] (Box 2). Infection may skew the immune response towards the Th2 pattern observed in allergy [61,106,107]. This effect appears to be mediated through changes in methylation [106,107,108]. Methylation levels at three CpG sites can separate (with area under the curve (AUC) = 1) children who will develop recurrent wheeze and asthma following an RSV infection from those who recover normally [108].

An epigenetic biomarker (Figure 2D) captures this interplay between environmental and genetic factors and could be beneficial for diagnosis at different stages of life, including for (1) newborns at high risk for asthma and other allergic diseases; (2) transient vs. persistent asthma at mid-childhood; (3) girls predisposed to severe asthma in adolescence and adulthood; and (4) pregnant women whose asthma may increase in severity (Box 3 and Figure 2D).

Box 3 **The importance of the epigenetic clock as a biomarker.**

Biomarkers are of essential importance for the timely and accurate diagnosis of asthma and allergic diseases [109]. DNA methylation may be involved in key aspects of the asthma phenotype, including age-related changes in presentation, prevalence, and severity. The epigenetic clock is a “higher order” summary of DNA methylation at key CpG sites [12]. It has been previously used as a biomarker in other complex conditions [109].

Epigenetic age is easy to assess using blood samples often collected during routine medical assessments. In addition, the epigenetic clock utilizes a small set of CpG sites rather than the whole genome, making it suitable for wide-spread use. While more studies are needed on epigenetic age acceleration in relation to asthma, the current literature has reliably shown associations between epigenetic age acceleration, asthma, and key lung characteristics (e.g., FEV_1_, lung capacity). These findings demonstrate exciting potential for the application of the epigenetic clock as a diagnostic marker for asthma, but more work is needed to validate this.

The epigenetic clock demonstrates the complex relationship between DNAm and a phenotype through its impact on aging. The few available studies show a positive association between allergy and asthma and epigenetic age acceleration [16,17]. Peng et al. found that extrinsic epigenetic age acceleration was linked to asthma and allergic disease (i.e., atopy, food allergy) in Project Viva—a longitudinal birth cohort with blood methylation data at mid-childhood (mean age: 7.8 years, range: 6.7–10.2 years) [16]. Both Horvath-predicted epigenetic age and intrinsic epigenetic age acceleration have also been associated with allergic disease [16]. These results were independently replicated in the Genetics of Asthma in Costa Rica Study (GACRS) cohort. Moreover, a study examining the methylation profiles of nasal epithelium cells from 547 children in early adolescence (mean age = 12.9) also showed increased epigenetic age in those with asthma [17].

Epigenetic age acceleration has also been linked to forced expiratory volume in one second (FEV_1_)—a key measure of lung fitness and asthma severity—in older and middle-aged individuals [110,111]. Both FEV_1_ and the ratio of FEV_1_ to forced vital capacity (FVC) were significantly negatively associated with epigenetic age acceleration. Epigenetic age has also been used to predict lung capacity in adults [112]. More investigations are needed into the relationship between FEV and epigenetic age, particularly in pediatric cohorts.

The epigenetic clock may provide a greater understanding of the sex-specific asthma prevalence between childhood and adulthood [31,113] (Box 4). Hormonal fluctuations during puberty, menstruation, pregnancy, and menopause may be associated with asthma pathogenesis, exacerbations, and disease severity [31] (Figure 2B,C and Box 4). This might explain the shift in asthma prevalence in adolescence [31] (Figure 2B). However, the impact of pregnancy is variable, with increased severity in some individuals and a decrease in others [114,115] (Box 4).

Sex hormones are key in the immune response [116]; thus, an epigenetic clock that captures early development and puberty may be crucial to understanding the relationship between EAA and asthma (Figure 2C, Box 1 and Box 4). A study by Patel et al. identified 13 CpG sites with sex-specific methylation associated with the acquisition of asthma between the ages of 10 and 18 [35]. Epigenetic clocks are also affected by sex and may unravel the relationships between DNAm, asthma, and sex [89,94] (Box 4).

Box 4 **Sex hormones, asthma, and the epigenetic clock.**

Asthma develops in an age- and sex-specific manner. During early childhood—when sex hormones are at low levels—both asthma and atopy are more common in boys [117]. This trend is reversed in adolescence [31,117]—a period of rapid increase in sex hormones—with more women becoming asthmatic [117].

Asthma symptoms are affected by hormonal fluctuations [118,119], with changes in severity noted during pregnancy and menstruation [117,120] and significant exacerbations at menopause [120]. Sex hormones modulate the immune response [116], a key driver of asthma [117,121]. Estrogen replacement therapy has been linked to the reactivation of asthma in menopausal women [122]. In mouse experiments, exposure to environmental estrogens led to an increase in a phenotype similar to asthma [123]. However, heterogeneity in the impact of hormones shows that other factors are also at play. Studies on oral contraceptives in asthma have produced contradictory results [124,125]. In addition, there is variability in the effects of pregnancy. One third of asthmatic pregnant women have milder symptoms of asthma [114,115] while pregnant, whereas another 1/3 experience exacerbations [114], and the remainder report no change. An epigenetic biomarker could help understand these differences. Epigenetic aging demonstrates sex-specific patterns, with higher epigenetic age acceleration observed in males throughout the lifespan [126,127] and may also be influenced by sex hormones [128,129,130]. Animal studies have shown that [129] castration (i.e., loss of testosterone) slows epigenetic age acceleration while loss of estrogen accelerates it [131]. In humans, intrinsic epigenetic age acceleration has been linked to age at menarche [128] and menopause [95], while hormone replacement therapy has been associated with slowing of epigenetic age acceleration [130].

## 13. Epigenetic Age Acceleration and the Developmental Origins of Health and Disease

Development is a highly complex process (Figure 2A). Environmental exposures may perturb methylation during this time, leading to long-term changes that influence susceptibility to disease. Low levels of DNAm across the genome at birth leave it vulnerable to aberrant methylation due to external factors (Figure 2A). These methylation patterns may persist and influence the epigenetic clock throughout the lifespan and across generations.

Previous studies have primarily focused on the prenatal and early life periods, but rapid change also occurs during later periods (e.g., puberty and even menopause). Smoking can alter methylation during adolescence [132], but whether this leads to impacts on epigenetic aging that continue in later life remains underexplored. Broader societal changes (e.g., industrialization) may also impact DNA methylation and, by extension, the epigenetic clock, which persists throughout the lifespan and across generations.

It has been suggested that an individual’s epigenetic age acceleration trajectory is established in childhood and continues at the same rate throughout life [133]. This question, as well as the possibility of the inheritance of age acceleration across generations, needs to be further examined.

Most current epigenetic clocks, except the PedBE clock and gestational age clocks, were developed using mainly adult samples and may miss sites involved in growth and developmental processes necessary to answer these questions. Moreover, the influence of environmental exposures during puberty, pregnancy, and menopause needs to be explored.

## 14. Conclusions

In this review, we have summarized prominent epigenetic clocks and their applicability to childhood asthma and allergic disease. These clocks have the qualities of a suitable diagnostic biomarker as they require data from only a small set of CpG sites from tissues such as blood and saliva that are routinely collected and can be easily accessed. The epigenetic clock bridges the relationship between genetic and environmental factors as well as the time-dependent course of asthma. The clock could have utility in the differentiation between transient and persistent asthma symptoms in childhood and the identification of at-risk individuals in adolescence and adulthood. As early life exposures drive asthma, understanding changes in DNA methylation during growth and development periods is of importance in refining the epigenetic clock as a pediatric asthma biomarker. Novel clocks incorporating pediatric longitudinal data can help further characterize the dynamic methylation patterns during these periods.

## Figures and Tables

**Table 1:** Examples of CpG sites associated with exposures or phenotypes in cord blood.

Publication	CpG Site	CHR	Position (hg38)	Gene	Associated Exposures or Phenotypes
[20]	cg14547404	10	48653753	*ARHGAP22*	Air Pollution
[20]	cg06517429	10	113679876	*CASP7*	Air Pollution
[21,25]	cg26995690	13	35772239	*DCLK1*	Birthweight
[21,25]	cg00637745	2	120739758		Birthweight
[21,25]	cg07133097	2	120739962		Birthweight
[19]	cg10593758	5	76952917	*CRHBP*	Elevated Maternal BMI
[19]	cg07621682	19	41321853	*CCDC97*	Elevated Maternal BMI
[21,22,26]	cg11932158	3	155704340	*PLCH1*	Gestational Age
[21,26]	cg18623216	3	155704181	*PLCH1*	Gestational Age
[21,26]	cg16103712	8	98011641	*MATN2*	Gestational Age
[21,26]	cg17133774	1	6138607	*CHD5*	Gestational Age
[21,26]	cg12713583	19	940724	*ARID3A*	Gestational Age
[21,26]	cg04347477	12	124517461	*NCOR2*	Gestational Age
[21,26]	cg08817867	17	19753241		Gestational Age
[21,26]	cg02001279	19	940967	*ARID3A*	Gestational Age
[21,26]	cg08412913	16	85395916	*DOCK6*	Gestational Age
[21,26]	cg06870470	19	11205091		Gestational Age
[21,24,27]	cg05549655	15	74726802	*CYP1A1*	Maternal Smoking
[21,23,27,28]	cg11924019	15	74726942	*CYP1A1*	Maternal Smoking
[21,23,27,28]	cg22549041	15	74726910	*CYP1A1*	Maternal Smoking
[21,23,24,27,28,29]	cg23067299	5	323791	*AHRR*	Maternal Smoking
[21,23,24,27]	cg22132788	7	44962886	*MYO1G*	Maternal Smoking
[21,23,27,28]	cg18092474	15	74726961	*CYP1A1*	Maternal Smoking
[21,23,24,27,28]	cg12803068	*7*	44963320	*MYO1G*	Maternal Smoking
[21,23,27]	cg12101586	15	74726862	*CYP1A1*	Maternal Smoking

Abbreviations: CpG; Cytosine-p-Guanine hg38; human genome version 38. Genes associated with the CpG site are indicated if available. For gestational age, DNAm associated with change in gestational age (in weeks).

**Table 2:** First-generation epigenetic clocks.

Type	Epigenetic Clock	Tissue Type	Methodology Used	Methylation Technology	Strengths	Limitations
Single Tissue	[83]	Single Tissue: Saliva	Association Analysis	Illumina 27 K Array	First epigenetic clock	Low accuracy (mean AE: 5.2 years)
	[79]	Single Tissue: Whole Blood	Elastic Net with bootstrapping	Illumina 450 K Array	Accurate in blood. Extensively used	Limited age range of training samples: 19–101 years
	[84]	Single Tissue: Whole Blood	Multivariate Linear Regression	Pyrosequencing	Consists of only three CpG sites	Low accuracy (mean AE: 5.4 years)
	[85]	Single Tissue: Breast Tissue	Elastic Net Regression with cross-validation	TruSeq Methyl Capture EPIC library	Improved accuracy in breast tissue	TruSeq Methyl Capture not yet widely used
Multi-Tissue	[86]	Multi-Tissue: Epidermis, dermis, T-cells, cervical smear, and monocytes	Pearson Correlation	Illumina 27 K Array	First multi-tissue clock	Relative low accuracy (mean AE: 11 years)
	[65]	Multi-Tissue: 51 tissue and cell types	Elastic Net with ten-fold cross-validation	Illumina 27 K Array and Illumina 450 K Array	Accurate across tissues; extensively used	Mostly adult samples Age of neonate samples set at “0”
	[80]	Multi-Tissue including blood	Elastic Net with ten-fold cross-validation	Illumina 450 K and Illumina EPIC Array	Accurate (mean AE: 2.5 years)	Not widely used yet
Pediatric Single Tissue	[81]	Single Tissue: Buccal Cells	Elastic Net with cross-validation	Illumina EPIC Array	Pediatric-only clock	Low accuracy in blood
Gestational Age	[87]	Cord Blood	Elastic Net Regression with cross-validation	Illumina 27 K array and Illumina 450 K Array	Median error: 1.24 weeks	Gestational Age Only
	[88]	Cord Blood	Lasso Regression with cross-validation	Illumina EPIC Array	Uses the EPIC Array	Gestational Age Only
	[26]	Cord Blood	Elastic Net Regression	Illumina 450 K Array	Correlation with Gestational Age	Gestational Age Only

**Table 3:** Second-generation epigenetic clocks.

Epigenetic Clock Citation	Tissue Type	Methodology Used	Platform	Strengths	Limitations
[77]	Elastic Net with Cross Validation	Phenotypic Age	Illumina EPIC Array	Composite of aging; well correlated with morbidity	Utility of childhood samples is unknown
[90]	Elastic Net with Cross Validation	Time-to-Death	Illumina 450 K and EPIC Arrays	Well correlated with mortality	Utility of childhood samples is unknown

## Data Availability

Not applicable.
